# LAT-1 and GLUT-1 Carrier Expression and Its Prognostic Value in Gastroenteropancreatic Neuroendocrine Tumors

**DOI:** 10.3390/cancers12102968

**Published:** 2020-10-13

**Authors:** Miguel Sampedro-Núñez, Antonio Bouthelier, Ana Serrano-Somavilla, Rebeca Martínez-Hernández, Magdalena Adrados, Elena Martín-Pérez, José Luis Muñoz de Nova, José Manuel Cameselle-Teijeiro, Concepción Blanco-Carrera, José Manuel Cabezas-Agricola, José Ángel Díaz, Rogelio García-Centeno, Julian Aragones, Mónica Marazuela

**Affiliations:** 1 Services of Endocrinology, Hospital Universitario de la Princesa, 28006 Madrid, Spain; miguelantonio.sampedro@salud.madrid.org (M.S.-N.); ana.serrano@salud.madrid.org (A.S.-S.); rebeca.martinez@salud.madrid.org (R.M.-H.); 2 Immunology and Molecular Biology Unit, Universidad Autónoma de Madrid, Instituto Princesa, 28006 Madrid, Spain; 3 Research Unit, Hospital of Santa Cristina, Research Institute Princesa (IP), Autonomous University of Madrid, 28009 Madrid, Spain; antonio.bouthelier@salud.madrid.org (A.B.); julian.aragones@salud.madrid.org (J.A.); 4 Service of Pathology, Hospital Universitario de la Princesa, Universidad Autónoma de Madrid, 28006 Madrid, Spain; mmagdalena.adrados@salud.madrid.org; 5 Service of Surgery and Digestive Surgery, Hospital Universitario de la Princesa, Universidad Autónoma de Madrid, Instituto Princesa, 28006 Madrid, Spain; emperez@salud.madrid.org (E.M.-P.); jmunoz@salud.madrid.org (J.L.M.d.N.); 6 Service of Pathology, Hospital Clinico Universitario, Universidad de Santiago de Compostela, 15706 A Coruña, Spain; josemanuel.cameselle@usc.es; 7 Service of Endocrinology, Hospital Universitario Príncipe de Asturias, Universidad de Alcalá de Henares, 28805 Madrid, Spain; concepcion.blanco@salud.madrid.org; 8 Service of Endocrinology, Hospital Clinico Universitario, Universidad de Santiago de Compostela, 15706 A Coruña, Spain; jose.manuel.cabezas.agricola@sergas.es; 9 Service of Endocrinology, Hospital Clinico San Carlos, Universidad Complutense de Madrid, 28040 Madrid, Spain; joseangel.diaz@salud.madrid.org; 10 Service of Endocrinology, Hospital Universitario Gregorio Marañón, 28007 Madrid, Spain; rogelio.garcia@salud.madrid.org; 11 CIBER de Enfermedades Cardiovasculares (CIBERCV), Carlos III Health Institute, 28029 Madrid, Spain

**Keywords:** neuroendocrine tumors, LAT-1, GLUT-1, biomarker, gastroenteropancreatic neuroendocrine tumors

## Abstract

**Simple Summary:**

Gastroenteropancreatic neuroendocrine tumors (GEP-NETs) represent about 70% of all NETs; however, improvement in their outcomes has yet to be achieved. Here, we aimed to analyze the role of metabolic players such as the amino acid transporter 1 (LAT-1) and glucose transporter 1 (GLUT-1), regulated by the oxygen-sensing mechanism Von Hippel Lindau-hypoxia-inducible factor (VHL-HIF), in gastroenteropancreatic neuroendocrine tumors (GEP-NET). We also aimed to correlate them with tumor malignancy and progression. We confirmed that specific mechanisms that increase nutrient uptake, such as LAT-1 and GLUT-1, are increased in GEP-NETs, whereas pVHL is decreased. Our results suggest that these biomarkers could have a potential role in NET pathophysiology which might be related to their proliferation and metastatic capacity.

**Abstract:**

Cancer cells develop mechanisms that increase nutrient uptake, including key nutrient carriers, such as amino acid transporter 1 (LAT-1) and glucose transporter 1 (GLUT-1), regulated by the oxygen-sensing Von Hippel Lindau-hypoxia-inducible factor (VHL-HIF) transcriptional pathway. We aimed to analyze these metabolic players in gastroenteropancreatic neuroendocrine tumors (GEP-NET) and correlate them with tumor malignancy and progression. LAT-1, GLUT-1, and pVHL expression was analyzed in 116 GEP-NETs and 48 peritumoral tissue samples by immunohistochemistry. LAT-1 was stably silenced using specific shRNA in the human NET BON cell line. LAT-1 expression was significantly increased in tumor tissue compared to non-tumor tissue in both gastrointestinal (67% vs. 44%) and pancreatic NETs (54% vs. 31%). Similarly, GLUT-1 was substantially elevated in gastrointestinal (74% vs. 19%) and pancreatic (58% vs. 4%) NETs. In contrast, pVHL expression was decreased (85% vs. 58%) in pancreatic NETs. Tumors with metastases at diagnosis displayed increased LAT-1 and GLUT-1 and decreased pVHL expression (*p* < 0.001). In accordance with these data, silencing LAT-1 curtailed cell proliferation in BON cells. These findings suggest that specific mechanisms that increase nutrient uptake, such as LAT-1 and GLUT-1, are increased in GEP-NETs, whereas pVHL is decreased. These markers might be related to the proliferation and metastatic capacity of these tumors.

## 1. Introduction

Gastroenteropancreatic neuroendocrine tumors (GEP-NETs) comprise an eclectic group of infrequent neoplasms that arise from enterochromaffin epithelial cells or from a variety of other neuroendocrine (NE) cells (e.g., alpha or beta cells, among others). Primary lesions of GEP-NETs tend to be found in the gastric mucosa, small and large intestine, rectum, and pancreas. GEP-NETs represent about 70% of all NETs [1] and, despite the rise in GEP-NET diagnoses, a concomitant improvement in outcomes has yet to be detected. Unlike other malignancies, the natural history of NETs is unpredictable and most well differentiated GEP-NETs remain indolent, even after the appearance of metastases, whereas others may progress rapidly with a median survival of 5–56 months [2]. Clinical guidelines have clearly defined disease stage by the tumor, lymph node, and metastasis (TNM) classification and tumor grade as the two major independent prognostic parameters for GEP-NET diagnosis [3,4]. While surgical resection of malignant tissue is the leading treatment option for GEP-NETs, a complete cure is rarely possible and several therapies are used, including targeted therapies (somatostatin analogues, everolimus, and sunitinib) and/or chemotherapy. Thus, characterization of biomarkers of prognosis and therapeutic response for these neoplasms is imperative [5].

Tumors require vast amounts of nutrients for biochemical reactions; consequently, a continuous supply is essential for their survival. The management of these nutrients is a crucial process that begins with their uptake from the extracellular space to the cytoplasm by specific transporters. Cancer cells generally overexpress some of these transporters in order to guarantee a massive influx of such nutrients to support their high proliferation rates. Given that amino acids (AA) are indispensable for tumor cell growth and metabolism, enhanced AA uptake is critical for tumor progression. Among the different essential amino acid (EAA) carriers, the large neutral amino acid transporter 1 (LAT-1, SLC7A5) is highly expressed in many cancer types. Thus, LAT-1 expression has been associated with tumor behavior and prognosis in certain cancers, including non-small cell lung cancer [6], prostate cancer [7], melanoma of the skin [8], breast cancer [9], gastric cancer [10], and renal cell carcinoma [11]. Some of these tumors are more related to metastases than others [12,13,14]. Likewise, LAT-1 is found in normal adults in highly proliferative tissues, including the gastrointestinal mucosa and pancreatic islet cells [15]; however, despite LAT constitutive expression in the intestine and pancreas, little is known about its role in GEP-NETs.

One of the cancer metabolic hallmarks is the switch from glucose oxidative phosphorylation to “aerobic glycolysis”, known as the Warburg effect [16]. This switch provides cancer cells with increased amount of energetic and anabolic supplies to support their extraordinary growth and invasiveness. GLUT-1 has received most of the attention within the glucose membrane transporter (GLUT) family, given its contribution to tumor glucose metabolism [17] and the fact that upregulation of GLUT-1 can substantially enhance glucose uptake and produce energy via accelerated glycolysis. GLUT-1 is overexpressed in various cancer types including breast cancer, gastric adenocarcinoma, sarcomas, lymphomas, melanomas, and hepatoblastomas [18,19,20]. GLUT-1 expression has likewise been linked to increased cell proliferation and tumor growth, promoting metastatic behavior, higher tumor grade, and poor clinical outcome [16]. GLUT-1 expression is increased in GEP-NETs and is significantly higher in grade 2 compared to grade 1 tumors [21]. A significant correlation between the Ki67 proliferation index and GLUT1 gene expression has been previously reported [22].

The expression of both LAT-1 and GLUT-1 transporters in tumor cells has been linked to hypoxia-inducible factors (HIFs). Indeed, HIF activation induces LAT-1 and GLUT-1 expression [11,17,23]. In normoxic conditions, HIFα subunits are hydroxylated by the prolyl-hydroxylases (PHD) allowing the binding of ubiquitin-ligase Von Hippel Lindau (VHL). In contrast, in hypoxic conditions, PHDs lack sufficient oxygen to hydroxylate the key proline residues leading to HIFα subunit stabilization. Mutations within the VHL gene disrupt the interaction between HIFα and pVHL, leading to constitutive HIF activation and expression of HIF targets involved in many cellular processes, including angiogenesis and cell metabolism [24,25]. Although VHL mutation is rare in sporadic pancreatic NETs, its inactivation is a significant pathway in the development of sporadic pancreatic NETs [26,27,28].

In this context, we sought to analyze the presence of metabolic players related to nutrient uptake and hypoxia such as LAT-1 and GLUT-1 in GEP-NETs and its possible relation with certain VHL mediated hypoxia pathways. We found that LAT-1 was overexpressed in tumor tissues compared to adjacent non-tumor tissue. Moreover, tumor cell proliferation was diminished upon LAT-1 silencing. Results from our study strongly suggest a potential role for LAT-1 in NET pathophysiology.

## 2. Results

### 2.1. Expression of LAT-1 and Other Angiogenesis Markersin GEP-NETs

We began by applying a tissue microarray (TMA) approach to investigate immunohistochemical expression of LAT-1, GLUT-1, and pVHL in a large set of GEP-NET tumor samples. For LAT-1 immunodetection, we used antibodies previously utilized for GLUT-1 and VHL immunodetection in paraffin sections [29,30] and a commercially available antibody (see Methods) capable of recognizing human LAT-1 in paraffin sections (Figure S1). LAT-1 expression was first assessed by immunohistochemistry (IHC) in tumor and peritumoral pancreatic or gastrointestinal tissue samples. We also validated LAT-1 expression by single cell immunofluorescence (Figure S2a) and flow cytometry (Figure S2a) in tumor cells from a single NET patient. Percentages of staining were calculated following previous criteria [31] and as described in Material and Methods.

**Figure 1:**
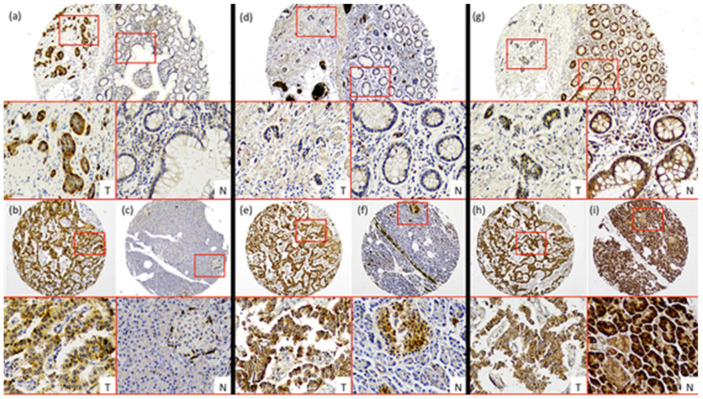
Characterization of nutrient transport markers in Gastroenteropancreatic neuroendocrine tumors (GEP-NETs). Representative cases of immunohistochemistry performed in a tissue microarray (TMA) of GEP-NET. (**a**–**c**) LAT-1 immunohistochemistry. (**d**–**f**) GLUT-1 immunohistochemistry and (**g**–**i**) pVHL immunohistochemistry. First row: intestinal tumors (**a**,**d**,**g**), tumor [T]/normal [N] zones with their respective magnification inset [T/N]. Second row: (**b**,**e**,**h**) pancreatic tumor and (**c**,**f**,**i**) normal tissue from the same tumor sample with their respective magnification inset [T/N]. Original magnification 4× with 20× digital zoom (insets).

**Figure 2:**
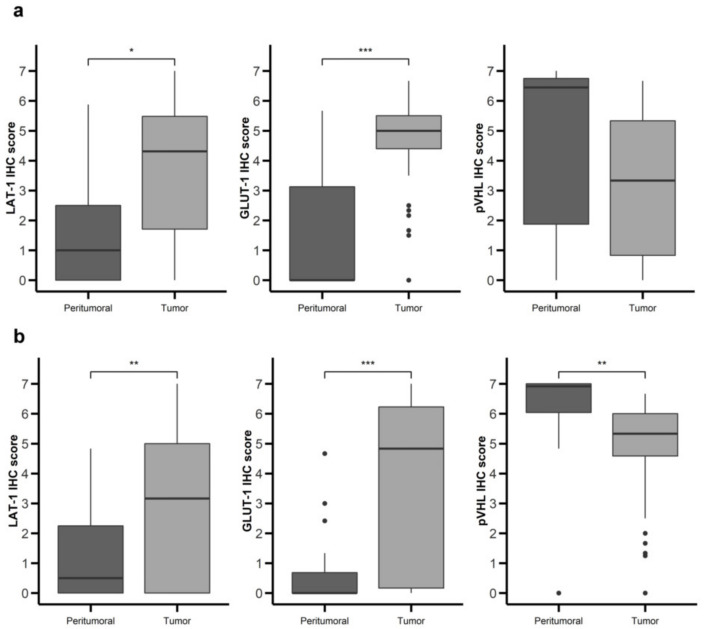
Nutrient transport markers are differentially expressed in GEP-NETs compared to normal adjacent tissue. LAT-1, GLUT-1, and pVHL were measured by immunohistochemistry (IHC) in a set of TMAs (*n* = 164) from GEP-NETs, including primary and metastatic tissue. Values are represented as boxplot of IHC scores in tumor tissue and in normal peritumoral tissue. (**a**) Comparison in gastroenteric NETs. (**b**) Comparison in pancreatic NETs. Boxplots visualize five statistics (the median, first quartile, third quartile, and two whiskers). The upper and lower hinges correspond to the third (75th percentile) and first (25th percentile) quartiles. The upper whisker extends from the hinge to the largest value no further than 1.5 fold the interquartile range (IQR) of the hinge. The lower whisker extends from the hinge to the smallest value at most 1.5 fold the IQR of the hinge. Asterisks indicate significant differences between tumor and peritumoral tissues (* *p* < 0.05, ** *p* < 0.01, *** *p* < 0.001).

LAT-1 staining showed a strong membranous signal in tumor cells (Figure 1a,b). Noteworthy, LAT-1 staining was more prominent in both gastroenteric (GE) (Figure 1a and Figure 2a) and pancreatic NETs (Figure 1b and Figure 2b) compared to adjacent, healthy, peritumoral tissue (67% vs. 44% and 54% vs. 31%, respectively; *p* < 0.05).

Similarly, the GLUT-1 signal was more conspicuous in both GE (Figure 1d and Figure 2a) (74% vs. 19%; *p* < 0.001) and pancreatic tumors (Figure 1e and Figure 2b) (58% vs. 4%; *p* < 0.001) than in healthy tissue. Immunodetection of LAT-1 and GLUT-1 was decreased in normal neuroendocrine (NE) cells from adjacent tissues with respect to NE cells from tumor tissues (Figure 1c,f). LAT-1 and GLUT-1 staining was only observed in sporadic Langerhans islet cells in normal pancreatic tissue and in sporadic crypt cells in normal intestinal tissues.

In contrast, the pVHL expression signal was weaker in tumor cells (Figure 1g,h) compared to NE cells from peritumoral or control samples (Figure 1g,i), in both GE (Figure 1g and Figure 2a) and pancreatic NETs (Figure 1h and Figure 2b) (38% vs. 67% and 58% vs. 85%, respectively; *p* < 0.01).

**Figure 3:**
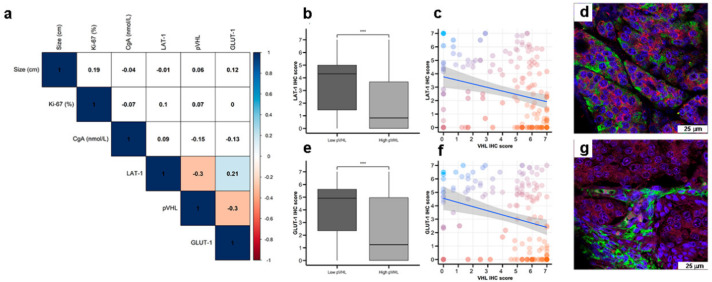
Co-expression of nutrient transport markers and VHL in GEP-NETs. LAT-1, GLUT-1, and pVHL were measured by IHC in a set of TMAs (*n* = 164) from GEP-NETs, including primary and metastatic tissue. (**a**) Correlation map of hypoxia marker expression in TMAs with tumor size and Ki67 and Chromogranin A (CgA). Values represent the Spearman’s rank correlation coefficient, rho (ρ). Significant negative correlations are shown in orange and significant positive correlations in blue. Color intensity increases with the magnitude of correlation. White indicates non-significant correlation. (**b**) Boxplot of LAT-1 IHC scores in low and high pVHL tumor tissues. (**c**) Scatter plot of LAT-1 and pVHL (regression line is represented with confidence interval). (**d**) Double immunofluorescence with LAT-1 (green) and pVHL (red) in intestinal tumor tissue. (**e**) Boxplot of GLUT-1 IHC scores in low and high pVHL tumor tissues. (**f**) Scatter plot of GLUT-1 and pVHL (regression line is represented with confidence interval). (**g**) Double immunofluorescence was performed with GLUT-1 (green) and pVHL (red) in intestinal tumor tissue. Scale bar for 75 µm is represented with a white box for each panel. Boxplots visualize five statistics (the median, first quartile, third quartile, and two whiskers). The upper and lower hinges correspond to the third (75th percentile) and first (25th percentile) quartiles. The upper whisker extends from the hinge to the largest value no further than 1.5 fold the interquartile range (IQR) of the hinge. The lower whisker extends from the hinge to the smallest value at most 1.5 fold the IQR of the hinge. Asterisks indicate significant differences between tumor and peritumoral tissues (*** *p* < 0.001).

Interestingly, LAT-1 and GLUT-1 expression correlated (Figure 3a, Spearman rho 0.21, *p* < 0.01). Furthermore, LAT-1 and GLUT-1 expression inversely correlated with pVHL expression (both Spearman rho −0.3, *p <* 0.001) in pancreatic and GE tumors (Figure 3b,c,e,f). In this regard, tumor cells that show higher signal for LAT-1 (Figure 3d) or GLUT-1 (Figure 3g) expression show less or not detectable signal for pVHL expression on dual immunofluorescence analysis. No correlation was found between expression of these molecules with a marker of NE differentiation (Chromogranin A) (Figure 3a).

### 2.2. LAT-1 Expression Associates with Increased GEP-NET Malignancy

**Figure 4:**
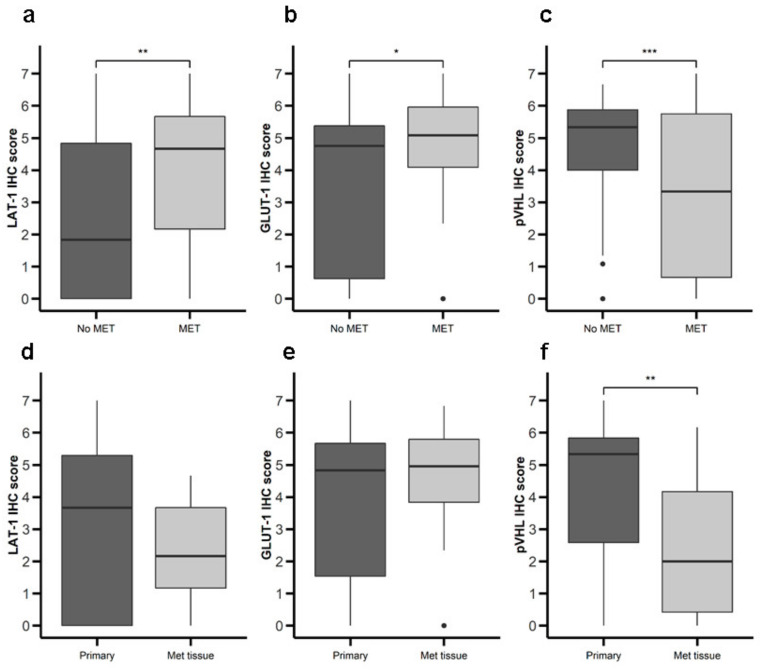
Expression of nutrient transport components and Von Hippel Lindau(VHL) in primary NETs with or without metastasis. LAT-1, GLUT-1, and pVHL were measured by IHC in a set of TMAs (*n* = 164) from GEP-NETs. Values are represented as boxplot of IHC scores. (**a**–**c**) Samples were classified based on metastatic status at the time of diagnosis: patients with metastasis (MET) or without metastasis (No MET). (**d**–**f**) Primary tumor tissue samples were compared to metastatic tumor tissue. Boxplots visualize five statistics (the median, first quartile, third quartile, and two whiskers). The upper and lower hinges correspond to the third (75th percentile) and first (25th percentile) quartiles. The upper whisker extends from the hinge to the largest value no further than 1.5 fold interquartile range (IQR) of the hinge. The lower whisker extends from the hinge to the smallest value at most 1.5 fold IQR of the hinge. Asterisks indicate significant differences between tumor and peritumoral tissues (* *p* < 0.05, ** *p* < 0.01, *** *p* < 0.001).

We proceeded to analyze the possible relationship between expression of LAT-1, GLUT-1, and pVHL and malignancy (Table 1). Primary tumor tissue from patients with metastatic disease at diagnosis exhibited greater LAT-1 (Figure 4a) and GLUT-1 (Figure 4b) expression and lower pVHL (Figure 4c) expression compared to tumors from patients with non-metastatic disease at diagnosis. Comparing primary to metastatic samples, we detected no difference in LAT-1 or GLUT-1 (Figure 4d,e), but found lower pVHL expression in metastatic tissue (Figure 4f). We did not find differences between LAT-1, GLUT-1, and pVHL expression associated with tumor grading and stage of disease (data not shown). No differences in survival rates were found comparing patients with high vs. low expression of any of the markers studied (data not shown).

### 2.3. Role of LAT-1 in NET Cell Proliferation

**Figure 5:**
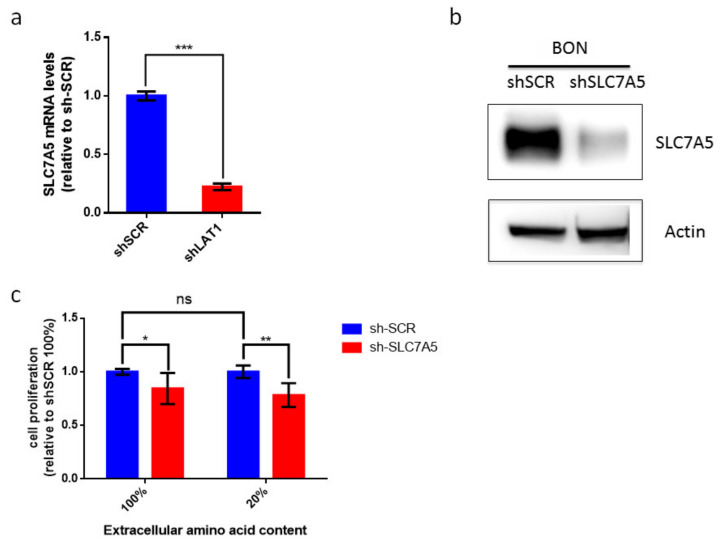
Contribution of LAT-1 (SLC7A5) to cell proliferation of BON tumor cell line. (**a**) Relative LAT-1 (SLC7A5) mRNA levels in sh-SCR and sh-SLC7A5 BON cells. Data are represented as the mean and error bars represent SEM (*n* = 3, *** *p* < 0.001). (**b**) Western blot analysis of SLC7A5 and actin protein levels in sh-SCR and sh-SLC7A5 BON cells. (**c**) BON sh-SCR and sh-SLC7A5 were cultured for 72 h in normal media (100%) or media containing 20% amino acid content. BON cell proliferation does not differ significantly between 100% and 20% amino acid content media. Proliferation of sh-SLC7A5 BON cells is expressed relative to sh-SCR BON cells in both amino acid content conditions. Data a represented as the mean and error bars represent SEM (*n* = 6) * *p* < 0.05, ** *p* < 0.01).

Our data suggest that LAT-1 (SLC7A5) expression can facilitate neuroendocrine tumor progression. The amino acid carrier LAT-1 is involved in the uptake of essential amino acids that subsequently leads to cell proliferation [11,32]. Consequently, we speculated that LAT-1 might control cell-autonomous proliferation in NETs. We therefore used the BON human cell line that derives from an enterochromaffin cell serotonin-producing pancreatic neuroendocrine tumor [33]. We first found that BON cells displayed basal mRNA and protein levels of LAT-1 (SLC7A5) (Figure 5a,b). Consequently, we generated BON-sh-SLC7A5 in which LAT-1 was silenced, as well as the corresponding BON-shScramble control cell line (Figure 5a,b; unprocessed Western blot images can be found in Figures S3–S5). Earlier studies have revealed that the impact of LAT-1 on tumor cell proliferation is evinced more clearly when extracellular amino acid content is reduced in cell culture media, thereby mimicking insufficient amino acid supply at the core of solid tumors [11,34,35]. Thus, we cultured BON-shSLC7A5 and BON-shScramble cells, not only in normal media (100% amino acid content), but also in media containing 20% of total amino acid content. Our data show that BON-shSLC7A5 cells proliferate less than BON-shScramble control cells in both amino acid content conditions, although the effect is greater in low amino acid content media (*p*-value < 0.05 and <0.01, respectively, Figure 5c).

## 3. Discussion

Tumor heterogeneity is one of the major impediments in studying GEP-NETs, precluding the detection of common molecular elements that will facilitate more effective diagnostic and therapeutic strategies. One approach to overcome these hurdles is to scrutinize the mechanisms that guarantee the availability of nutrients to tumor cells [36]. This is the first study to examine LAT-1 and GLUT-1 expression and its clinical significance in GEP-NETs. Our data suggest that both markers are highly expressed in pancreatic and GE cancer cells compared to non-malignant cells, and that both carriers could be explored in the future as potential predictive factors of outcomes in patients with a surgically resected GEP-NET.

It has been suggested that LAT-1 may participate in metastasis and several studies have unveiled a correlation of increased LAT-1 expression with metastasis and worse prognosis in bile duct adenocarcinomas, clear renal cell carcinoma, prostate cancer, and gastric cancer [37,38,39,40]. Interestingly, we detected that LAT-1 expression was associated with more aggressive tumors that had already metastasized at the time of diagnosis. These results indicate that LAT-1 overexpression may be a potential prognostic factor in GEP-NETs. In this regard, previous studies in pulmonary NETs have shown correlation between LAT-1 expression and tumor aggressiveness [41]. Furthermore, downregulation of LAT-1 expression inhibits gastric cell growth, migration, and invasion [10]. In this work, we have found novel data suggesting that restricting amino acid availability by decreasing LAT-1 expression could reduce cell proliferation in the NET BON cell line. Along this line, previous studies have shown that LAT1 silencing leads to a reduction of in vitro cell proliferation in different cell human lines, including renal cell carcinoma and colon adenocarcinoma cell lines. In addition, LAT silencing inhibits in vivo xenograft formation in mice [11,14,34]. Therefore, we believe that our data are strong evidence of the functional role of LAT in NETs with an impact in an “in vivo” biological setting. Based on these findings, LAT-1 inhibition could be considered a potential strategy for metastasis prevention in patients with a more aggressive disease (i.e., grade 3 neuroendocrine tumors), which is usually more dependent on amino acid and glucose metabolism.

Regarding GLUT-1, previous studies have reported several associations between GLUT-1 expression, tumor aggressiveness, and poor prognosis in other malignant neoplasms, including colorectal cancer, pancreatic ductal adenocarcinoma, lung cancer, prostate cancer, and [42,43,44,45,46]. One explanation is the increased utilization of energy and faster cell growth that indirectly promote metastatic behavior. GLUT-1 expression has been proposed in a recent meta-analysis as an independent prognostic marker for cancer in a wide range of tumors [47]. Furthermore, these data suggest that GLUT-1 expression is a potential prognostic marker able to guide treatment decisions. In a recent small study with lung neuroendocrine tumors, GLUT-1 showed a positive predictive value for the diagnosis of these tumors and was a useful tool for classifying pulmonary NETs [48].

Expression of cell glucose and AA transporters in tumor cells has been used for molecular imaging using transporter-specific PET probes. This technique has been used to diagnose location and/or activity of different tumors. Glucose metabolism is predominantly mediated by GLUT-1 transmembrane transporters [49] and represents the molecular basis underlying the use of radiolabeled glucose in FDG-PET. The rationale for using 18F-fluoroDOPA PET imaging is based on the fact that some tumors, including certain GEP-NETs, are able to take up, decarboxylate, and store amino acids and their biogenic amines such as L-DOPA, which is taken up by a LAT transporter [50]. These imaging techniques may be helpful as indirect markers of GLUT-1 (FDG) and LAT-1 (FDOPA) tumor expression. In this regard, GLUT-1 expression correlates with PET-FDG signal in pulmonary NETs [51]. Unfortunately, our cohort did not include enough PET studies to evaluate correlation of PET signal with LAT-1 and/ or GLUT-1 expression. This novel approach will likely help optimize patient treatment by improving monitoring of tumor grade staging, indicating the best therapeutic strategy, and monitoring response to therapy [52].

Increasing evidence indicates that tumors are able to use hypoxic stress to their benefit by activating key biochemical and cellular pathways for their progression, survival, and ability to metastasize. In this regard, we have previously reported a relationship of overexpression of angiopoietins, the angiopoietin TIE-2 receptor, and vascular endothelial growth factor (VEGF) with increased risk of metastasis in NETs [53]. Stress/hypoxia may play an important role in cell proliferation by stimulating or inhibiting intracellular pathways, such as HIF, that can modulate gene expression of multiple factors involved in nutrient uptake [54]. In normoxic conditions, the HIF pathway is inhibited, while the HIF2a isoform is activated [11,55]. Given that LAT-1 expression is controlled by HIF2a, this regulation might account for elevated LAT-1 expression, not only in hypoxic areas, but also in oxygenated cells, such as in pVHL-null cells, in which the HIF instability mechanism is disrupted [11,56]. In this context, individuals with non-hereditary (sporadic) pancreatic NETs may display functionally relevant VHL inactivation (haploinsufficiency or other kinds of epigenetic inactivation). This suggests that VHL gene alteration and the consequent effect on hypoxia signaling could condition the development of sporadic pancreatic NETs [26,27,28]. Interestingly, we found an inverse relationship between LAT-1 and GLUT-1 expression and VHL in GEP-NETs, indicating a possible disruption of the HIF pathway in these tumors.

We are aware that a possible weakness of the study is the lack of an ideal control to compare with the results of neuroendocrine intestinal tumors, due to the cellular heterogeneity of the intestinal crypts and a lower proportion of neuroendocrine cells. However, the control used in our study appears to be adequate, insofar as it included normal cells subjected to identical biological stress and processing to their respective tumor cell sample. The consistency of the results of this intestinal group with the results obtained in the pancreatic neuroendocrine tumor group provides additional support to the validity of our conclusions.

In this context, we propose that the study of markers of nutrient uptake at the tumor and peritumoral level, specifically LAT-1 and GLUT-1, could provide innovative knowledge regarding the complicated network of interactions in the NET cellular microenvironment. Therefore, the ability to modulate cellular uptake of glucose and AAs by means of GLUT-1 and LAT-1 inhibitors is currently deemed as a promising approach for the development of innovative anticancer therapies, which has thus far led to the discovery of several chemical classes of GLUT-1 and LAT-1 inhibitors. Interestingly, LAT-1 and GLUT-1 inhibiting drugs are currently in the pipeline and hold promise as both direct targets and factors to synergize with adjuvant therapies [57]. This basic knowledge might aid in personalizing drug regimens for these tumors in the future.

## 4. Materials and Methods

### 4.1. Individuals

A retrospective study was performed including consecutive patients with gastrointestinal and pancreatic NETs with available tumor samples from five reference centers in Spain (Hospital Universitario La Princesa, Hospital Clínico Universitario de Santiago de Compostela, Hospital Universitario de Alcalá de Henares, Hospital Clínico San Carlos, and Hospital Universitario Gregorio Marañón) between 1995 and 2018. Tumor samples from the same cohort have been previously reported [31]. Briefly, 110 patients with GEP-NETs were studied (62 had gastrointestinal NETs and 48 had pancreatic NETs) (Table 1). All participants were carefully screened for other malignancies and/or genetic disorders. One subject carried an MEN1 (multiple endocrine neoplasia type 1) gene mutation. No other apparent genetic abnormalities were found. A complete work-up including history, physical examination, and hormone levels was performed in all cases and interpreted by expert endocrinologists (M.M., C.B.C., J.C.A., J.A.D., R.G.C., and M.S.N.), classifying all patients according to World Health Organization (WHO) criteria (tumor site and size, angioinvasion, infiltration level, cell proliferation index, immunohistochemical phenotype, and metastases). All tumors were reviewed by the same pathologist (M.A) and were classified according to WHO criteria [58] as G1, G2, and G3 (Table 1). Cell proliferation activity was determined in terms of the Ki-67 index.

Subjects were managed following current recommendations and guidelines [59]. Elective surgery was the first treatment option in all cases and adjuvant therapy with somatostatin analogues was administered if evidence of residual disease was observed (Table 1).

This project was approved by the Internal Ethical Review Committee of the Hospital de La Princesa (Registration number: PI-776) and written informed consent was obtained from all participants prior to inclusion, in accordance with the Declaration of Helsinki.

### 4.2. Tissue Samples

A total of 164 formalin-fixed, paraffin-embedded tissues were evaluated by tissue microarray (TMA). Of them, 116 were tumor samples with pathological diagnosis and 48 were peritumoral. All samples were taken and managed in accordance with local regulations with the approval of the local Institutional Review Board.

### 4.3. Immunohistochemistry (IHC)

Tissue sections were treated by IHC as previously described [31]. Briefly, tissue sections were dewaxed, rehydrated, and washed in phosphate buffered saline 1X (PBS; Lonza, Basel, Switzerland). Epitope retrieval was performed by treating the slides in a PT Link (Dako, Santa Clara, CA, USA) containing an acid or basic solution. Next, endogenous peroxidase was inhibited with a peroxidase-blocking solution (Dako) for 10 min; Fc receptors were then blocked with goat or rabbit serum, as appropriate. Afterwards, sections were immunostained with the following primary antibodies: anti-Ki-67 (clone MIB-1, Dako), anti VHL (ref: sc135657; Santa Cruz Biotechnology, Dallas, TX, USA), anti-human CD98 light chain (LAT-1, SLC7A5, ref: AHP1139 Bio-Rad, Hercules, CA, USA), and anti-glucose transporter GLUT-1 antibody (ref: ab15309; Abcam, Cambridge, UK).

Next, sections were incubated with the proper horseradish peroxidase-conjugated secondary antibodies: goat anti-mouse (Ref: P0447, Dako), goat anti-rabbit (Ref: P0448, Dako), or rabbit anti-goat (Ref: P0449, Dako). Finally, sections were incubated with 3,3′-diaminobenzidine (DAB; Dako), counterstained with hematoxylin (Sigma-Aldrich, San Luis, AZ, USA), dehydrated in alcohol, cleared with xylene, and mounted. For each section, the approximate percentage of neuroendocrine (NE) positive cells (proportion score, PS) and staining intensity (intensity score, IS) determined the staining score (IHC score). In the case of pancreatic NETs, tumor cells were compared to neuroendocrine cells (NE) from the islets of Langerhans. In the absence of normal solid NE cell nests as a control for intestinal NET tumors, we used normal intestinal crypts with scattered epithelial neuroendocrine (NE) cells as a normal control for this subgroup. Five different high-power fields in the hot-spot areas of each slide underwent observer-blind examination by four independent observers (AS; MASN, MA, JMC). The proportion of stained cells in each field were scored following criteria as previously described [31]: 1, for 5% stained cells; 2, for 6–25% stained cells; 3, for 26–50% stained cells; and 4, for >50% stained cells. Overall stain intensity was graded as: 0, for negative staining; 1, for light staining; 2, for moderate staining, and 3, for intense staining. The total staining score (TS) for each field resulted from adding the proportion of stained cells score with the staining intensity score (TS = PS + IS). The final TS was the mean of the 5 fields. Tonsil tissue served as positive control for the different antibodies. Tumor expression of the various markers was classified with respect to the median as high (higher than or equal to the median) and low (lower than the median).

Immunostaining of agarose-embedded HEK 293T cells was performed by fixing cells in zinc formaline fixative (Ref: Z2902, Sigma-Aldrich) for 16 h. HEK 293T cells were then embedded in 1% agarose and prepared for paraffin processing. Paraffin slides underwent antigen retrieval by incubation in pH6 citrate buffer with heat induced epitope retrieval (HIER) conditions. Next, endogenous peroxidase was inhibited with 3% hydrogen peroxide for 15 min, followed by incubation with goat serum to block Fc antibody region. Subsequently, sections were immunostained with anti-LAT-1 (CD98 light chain) (Ref: AHP1139, Bio-Rad) followed by incubation with horseradish peroxidase-conjugated secondary antibody: polyclonal goat anti-rabbit HRP (Ref: P0448, Dako). Finally, sections were incubated with 3,3′-diaminobenzidine (DAB; Dako), dehydrated in alcohol, cleared with citrus clearing solvent (Ref: 8301 Thermo Scientific, Waltham, MA, USA), and mounted.

### 4.4. Immunofluorescence

Tissue sections were dewaxed, rehydrated, and washed in PBS 1X (Lonza). To retrieve epitopes, slides were treated in a PT Link (Dako) as described above for IHC. Sections were then incubated for 1 h with a blocking solution consisting of 2% (*w*/*v*) bovine serum albumin (BSA; Gibco) and 10% (*v*/*v*) human serum (Sigma-Aldrich) diluted in PBS × X as previously reported [31]. Sections were subsequently incubated overnight at 4 °C with the following primary antibodies: anti-CD3 (polyclonal, ref: A0452; Dako, Agilent Technologies, Santa Clara, CA, USA), anti VHL (ref: sc135657; Santa Cruz Biotechnology), anti-human CD98 (LAT-1, SLC7A5, ref: AHP1139; BioRad, Hercules, CA, USA), anti-glucose transporter GLUT-1 antibody (ref: ab15309; Abcam, Cambridge, UK), and chromogranin A antibody Alexa Fluor 488 (ref: NBP2-33198AF488; Novus Biological, CO, USA). The next day, tissue samples were washed three times for five minutes each with PBS 1X. Tissue sections were then incubated for 30 min with 4′,6-diamidino-2-phenylindole (DAPI, Thermo Fisher Scientific) and the appropriate secondary antibodies labeled with a fluorophore: donkey anti-rabbit Alexa Fluor 647 (Cat. A-31573, Invitrogen, Carlsbad, USA) or donkey anti-mouse Alexa Fluor 555 (Cat. A-31570, Invitrogen). Finally, sections were washed three times for five minutes each with PBS 1X, mounted, and analyzed with a Leica TCS-SP5 confocal microscope (Leica Microsystems, Wetzlar, Germany).

### 4.5. Cells and Reagents

All cells were maintained in Dulbecco’s modified Eagle’s medium (DMEM/HIGH GLUCOSE, SH30022.01, HyClone, GE Healthcare, Logan, UT, USA) and Ham’s nutrient mixture F1 medium (SH30026FS, HyClone, GE Healthcare) 1:1 supplemented with 100 units/mL penicillin, 100 μg/mL streptomycin, 20 mM HEPES, and 10% fetal bovine serum (FBS, SV30160.03 HyClone, GE Healthcare) as previously described [11]. To prepare media with lower amino acid content, normal DMEM/Ham’s F12 medium was diluted with the corresponding volume of amino acid-free DMEM. Amino acid-deprived media were supplemented with 10% dialyzed FBS (10,000 Mwt cut-off; F0392, Sigma-Aldrich).

### 4.6. Viral Infection

SLC7A5 (sc-62555-V) and control (sc-108080) shRNA lentiviral particles (Santa Cruz) were used to generate stable transfectants sh-SLC7A5 BON cells and their corresponding control sh-SCR BON cells based on our previous work. We took into consideration that pooling siRNAs that target different regions of the target mRNA has been proposed to diminish the off-target effect [60]. Therefore, we have used a commercially available pool of three shRNA constructs against SLC7A5 (sc-62555-V, Santa Cruz Biotechnology Inc.) as our strategy to silence SLC7A5 gene expression in the BON cell line. Lentiviral infection was conducted by seeding HEK293T cells in p60 plates transfected with 1.8 µg of each lentiviral vector 1.14 µg of pCMV-dR8.91 and pMD2.G using lipofectamine 2000 (Invitrogen), as per the manufacturer’s instructions. Cell culture supernatants were harvested 48 h following transfection, filtered through a 0.45-µm filter, diluted (1:2) with fresh medium containing 8 µg/mL polybrene (final concentration), and added to BON cells. This step was repeated over the following 2 days.

### 4.7. Cell Proliferation Analysis

For cell proliferation assays, 75,000 BON cells were seeded per well in 6-well plates. After 72 h, they were trypsinized, collected in the medium, and live cells per well were analyzed with methyl violet and measured by spectrophotometry at 540 nm. For cell proliferation recovery assays, 6 h after seeding, the medium was changed to complete medium or 20% medium and supplemented with dialyzed FBS. Then cells were grown for another 72 h and collected and counted as previously described.

### 4.8. Western Blot and Antibodies

Western blot was performed using the same methods as previously reported [11], albeit a different antibody for LAT-1 detection was used (cell signaling, #5347). Specifically, cells were lysed in Laemmli buffer. Western blots were performed using 8–12% SDS-polyacrylamide gels and the membranes were probed with rabbit polyclonal antisera raised against LAT-1 (cell signaling, #5347) or β-actin (A3854, Sigma-Aldrich). Immunolabeling was detected by enhanced chemiluminescence (SuperSignal West Femto Maximum Sensitivity Substrate, Thermo Scientific) and visualized with a digital luminescent image analyzer (Image Quant LAS400 mini).

### 4.9. RNA Extraction and qRT-PCR

Total RNA from cells was isolated using Ultraspec and TRIsure (BIO-38032, Bioline). RNA (1 µg) was then reverse-transcribed with Improm-II reverse transcriptase (Promega) and polymerase chain reaction (PCR) amplifications were performed using the Power SYBR Green PCR Master Mix kit (Applied Biosystems) in a StepOne Version 2.0 system (Applied Biosystems) [11]. The following primer sets were utilized: human LAT-1 (forward, 5′-GGAACATTGTGCTGGCATTATACA-3′; reverse, 5′-CCTCTGTGACGAAATTCAAGTAATTC-3′), and human 28s (forward, 5′-GGTAGCCAAATGCCTCGTCAT-3′; reverse, 5′-GGATAGTAGGTAGGGACAGTGGGAAT-3′). LAT-1 RNA data were normalized to 28S RNA expression levels. Moreover, LAT-1 and 28S RNA data were obtained using a standard curve for both LAT-1 and 28S. Data were analyzed with Software StepOne version 2.0 (Applied Biosystems, available from: https://www.thermofisher.com/es/es/home/technical-resources/software-downloads/StepOne-and-StepOnePlus-Real-Time-PCR-System.html).

### 4.10. Statistical Analysis

Quantitative variables are expressed as the median and interquartile range (boxplots), while qualitative variables are presented as relative percentages of samples (histograms) included in contingency tables. Fisher’s exact test was applied to compare qualitative variables. Unpaired, two-tailed, Student t-test was used to compare two independent groups and paired Student t-test, to analyze two related samples. One-way ANOVA was employed to compare more than two groups and post-hoc multiple comparisons were made with Tukey’s test. Spearman’s rho analyses were performed to detect correlations between blood markers and immune markers examined by IHC. Kaplan–Meier analyses were used to assess the effect of the different biomarkers on cohort survival. Stata v. 12.0 (StataCorp. 2011, College Station, TX, USA) for Windows and R version 3.3.2 [61] were used for analyses. Package ggplots2 [62] and corrplot (available from: https://github.com/taiyun/corrplot) were used for graphics. *p*-values < 0.05 were deemed statistically significant. * *p* < 0.05, ** *p* < 0.01, and *** *p* < 0.001.

## 5. Conclusions

We have shown that mechanisms that increase nutrient uptake, such as LAT-1 and GLUT-1, are increased in GEP-NETs, whereas pVHL is decreased in these tumors. Furthermore, tumors with metastases at diagnosis display a higher staining score of LAT-1 and GLUT-1 and a decreased pVHL expression suggesting a possible use of these markers as disease predictors in the future. Finally, functional studies suggest that LAT-1 disruption in NET BON cell line modify in-vitro proliferation capacity. In this context, our data suggest that LAT-1 inhibitors may play a role in the treatment of high-risk patients.

## Figures and Tables

**Table 1:** Patients’ baseline characteristics (*n* = 110) and sample characteristics (*n* = 164).

Gender	Number of Patients (Percentage)
Male	47 (42.7%)
Female	63 (57.3%)
**Age, years (at diagnosis)**	
<55	46 (41.8%)
≥55	64 (58.2%)
**Stage (ENETS at diagnosis)**	
I	32 (29.1%)
II	20 (18.2%)
III	19 (17.3%)
IV	36 (32.7%)
Unknown	3 (2.7%)
**Primary site**	
Pancreatic NET	48 (43.6%)
Gastroenteric NET	62 (56.4%)
**Primary tumor size, cm**	
<3.0	69 (62.7%)
≥3.0	37 (33.7%)
Unknown	4 (3.6%)
**Grading (WHO 2010 criteria)**	
G1	66 (60%)
G2	34 (30.9%)
G3	4 (3.6%)
Unknown	6 (5.5%)
**Sample characteristics (*n* = 164)**	
Primary tumor tissue	104 (63.4%)
Metastatic tumor tissue	12 (7.3%)
Adjacent non-tumor tissue	48 (29.3%)

Abbreviations: TNM: tumor, lymph node, metastasis; ENETS: European Neuroendocrine Tumor Society; NET: neuroendocrine tumor; WHO: World Health Organization; G1: Grade 1; G2: Grade 2; G3: Grade 3.

## Supplementary Materials

The following are available online at https://www.mdpi.com/2072-6694/12/10/2968/s1, Figure S1: LAT-1 (SLC7A5) immunodetection by immunohistochemistry and flow cytometry. Figure S2: FACs antibody validation on flow cytometry and immunofluorescence. Figure S3: Unprocessed western blot images of SLC7A5 and actin protein levels in sh-SCR and sh-SLC7A5 BON-1 cells presented in Figure 5b. Densitometry readings and ratio of SLC7A5/Actin of each band. Figure S4: Unprocessed western blot images of actin protein levels in sh-SCR and sh-SLC7A5 BON-1 cells presented in Figure 5b at different exposure levels. Figure S5: Unprocessed western blot images of SLC7A5 and actin protein levels in sh-SCR and sh-SLC7A5 BON-1 cells presented in Figure 5b. 25 to 70 kDa membrane photo.
