# Supplementary material for: LAT-1 and GLUT-1 Carrier Expression and Its Prognostic Value in Gastroenteropancreatic Neuroendocrine Tumors
Source: Cancers (Basel). 2020 Oct 13;12(10):2968. doi: 10.3390/cancers12102968 (PMC7602091; doi:10.3390/cancers12102968)
Supplement: Supplementary file 1 [file cancers-12-02968-s001.pdf]

cancers

Supplementary Materials: LAT-1 and GLUT-1 carrier expression and its prognostic value in gastroenteropancreatic neuroendocrine tumors

Sampedro-Núñez Miguel, Bouthelier Antonio, Serrano-Somavilla Ana, Martínez-Hernández Rebeca, Adrados Magdalena, Martín-Pérez Elena, Muñoz de Nova José Luis, Cameselle-Teijeiro José M, Blanco-Carrera Concepción, Cabezas-Agricola José Manuel, Díaz José Ángel, García-Centeno Rogelio, Aragones Julian and Marazuela Monica.

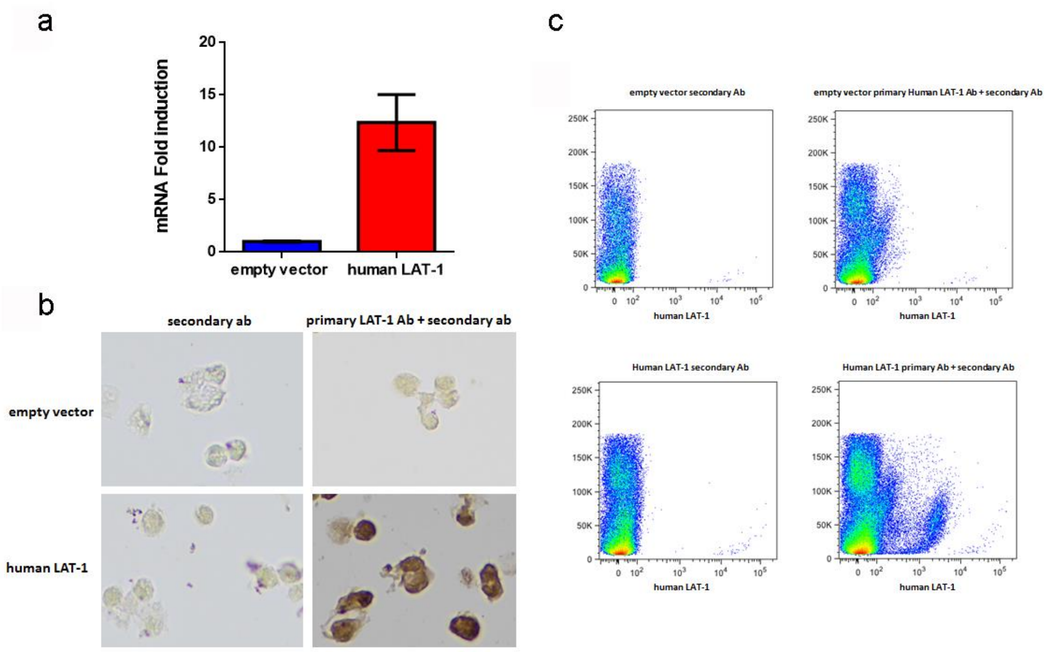

Figure S1. LAT-1 (SLC7A5) immunodetection by immunohistochemistry and flow cytometry.

(a) Relative LAT-1 mRNA levels in HEK 293T transfected with control empty vector or human LAT-1 expression vector. The mean is shown and error bars represent SEM (n=5, ***p <0.008). **(b)** Representative immunohistochemistry pictures of HEK 293T transfected with control empty vector or human LAT-1 expression vector incubated with secondary antibody goat α-rabbit HRP or primary LAT-1 antibody and secondary goat α-rabbit HRP. **(c)** Flow cytometry dot plot representation of HEK 293T transfected with control empty vector or human LAT-1 expression vector stained with secondary antibody donkey α-rabbit APC or primary LAT-1 antibody and secondary donkey α-rabbit APC (f).

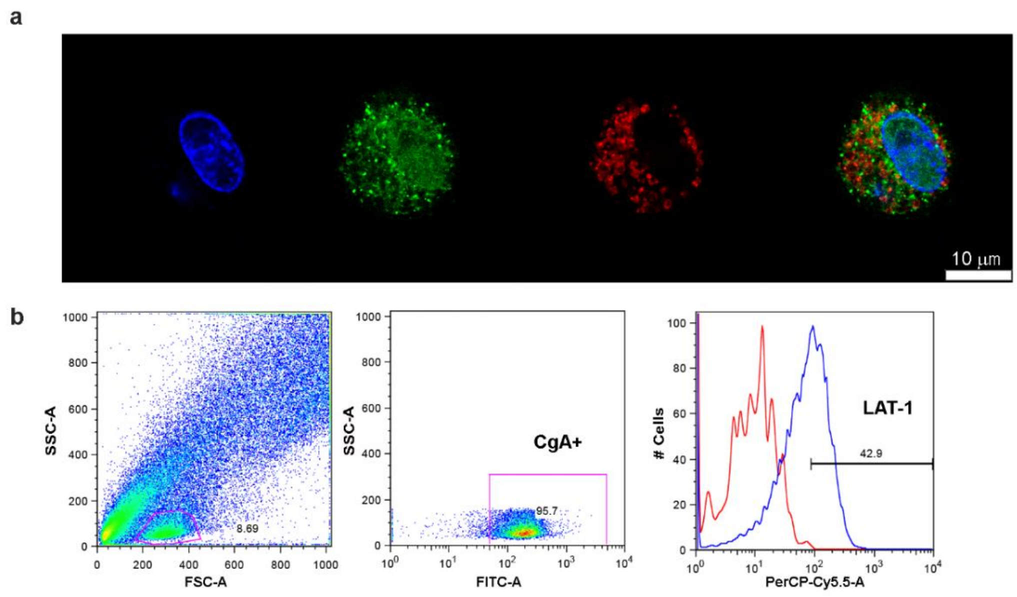

Figure S2. FACs antibody validation on flow cytometry and immunofluorescence.

(a) Singles Cell Dual immunofluorescence label with CgA (green) and LAT-1 (red). Scale bar is represented with a white line in the merge panel. **(b)** Dot plot of tumor cells from a representative GEP-NET patient. Most tumoral cells stained with CgA (stained with FITC fluorophore, 95.7% - middle panel); some of these cells also expressed LAT-1 (stained with PerCp fluorophore, 42.9% - right panel). Red histogram plot data on negative control while blue histogram plots data on marked sample.

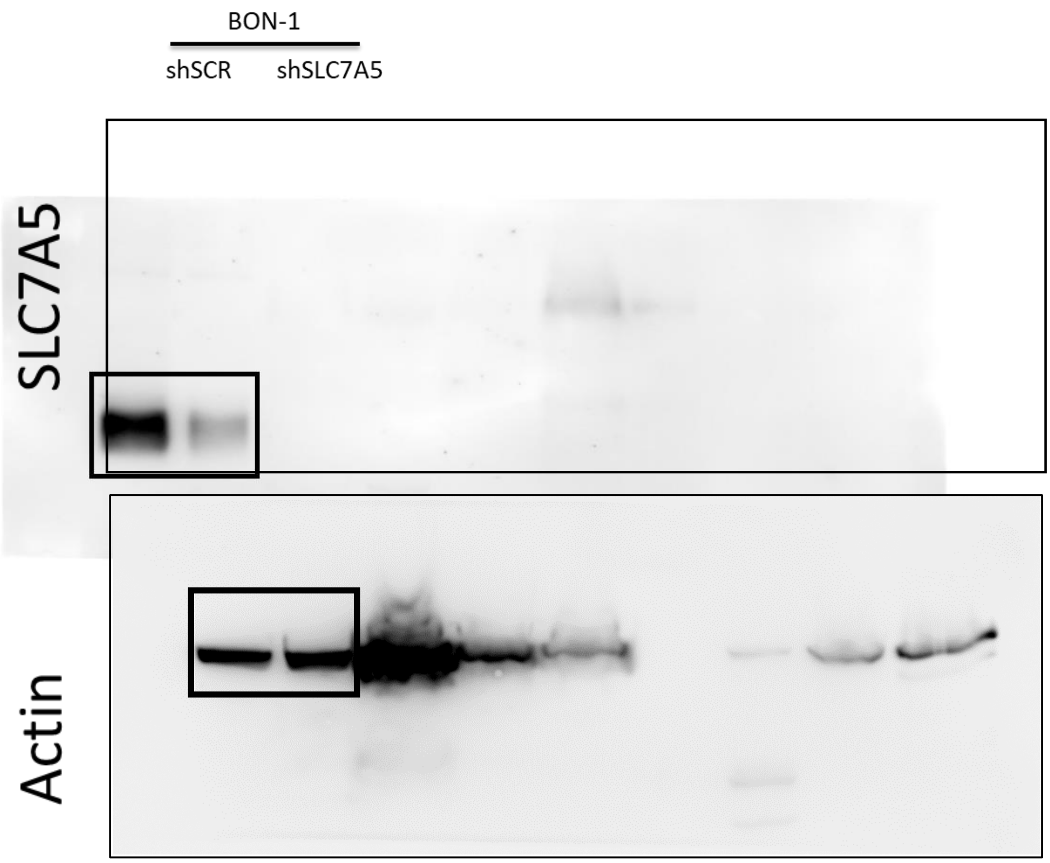

	shSCR	shSLC7A5
**SLC7A5**	24291.94	5918.22
**Actin**	12207.63	16271.87
**ratio SLC7A5/Actin**	1.98989812	0.36370866

Figure S3. Unprocessed western blot images of SLC7A5 and actin protein levels in sh-SCR and sh-SLC7A5 BON-1 cells presented in Fig. 5b. Densitometry readings and ratio of SLC7A5/Actin of each band.

Actin exp: 0,125s.

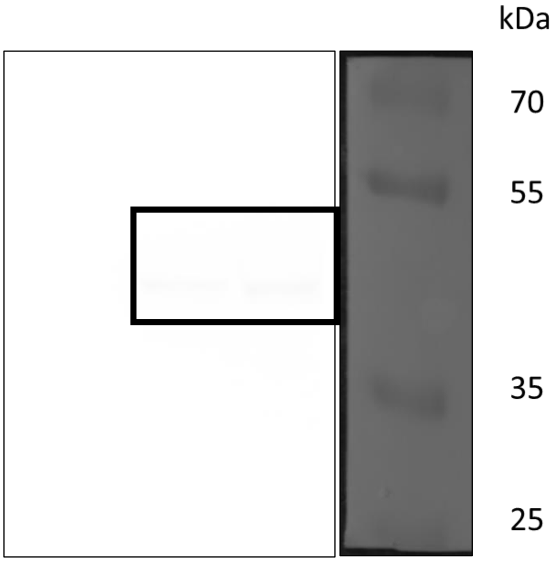

Actin exp:1s.

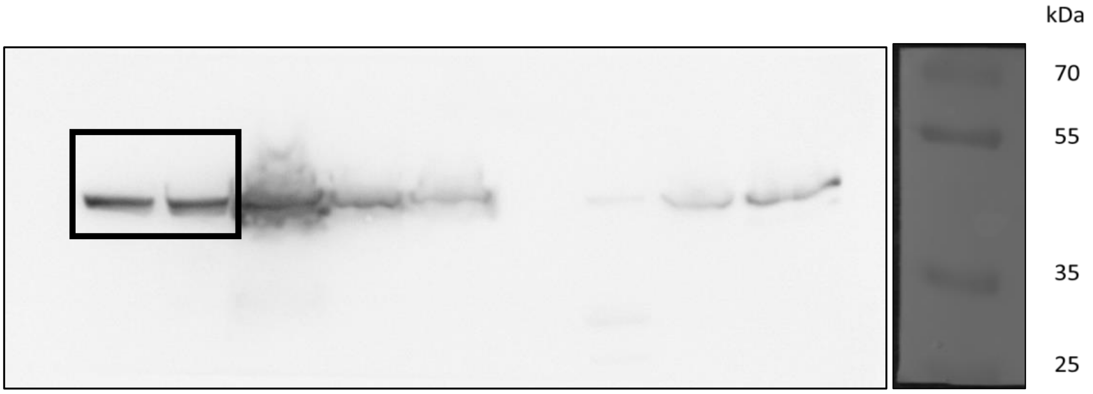

Actin exp:3s.

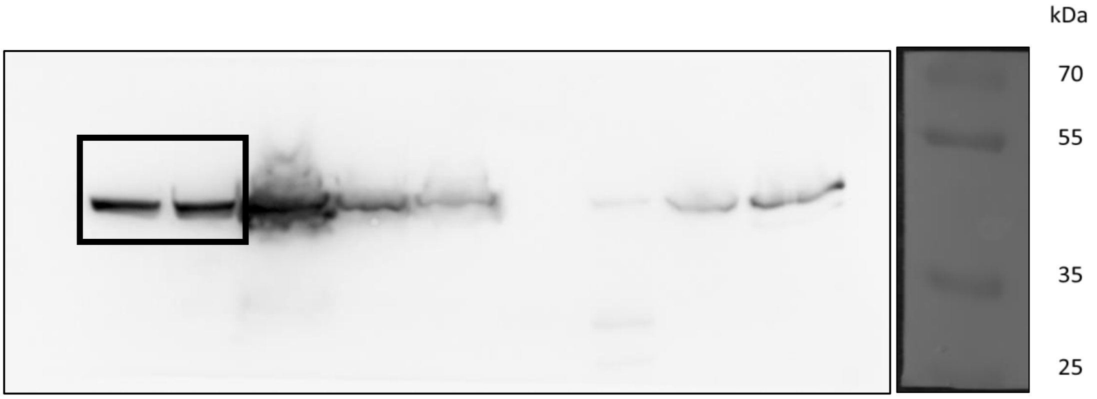

Actin exp:7s.

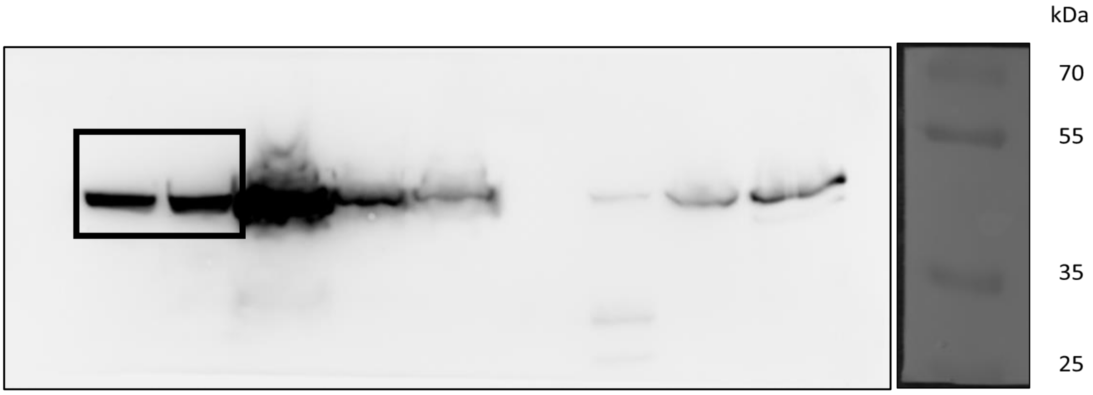

Actin exp:15s.

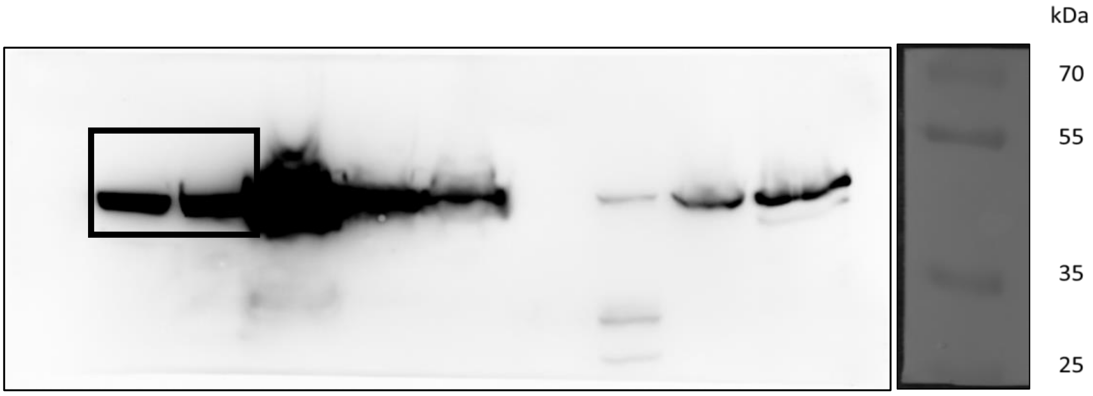

SLC7A5 exp:5s.

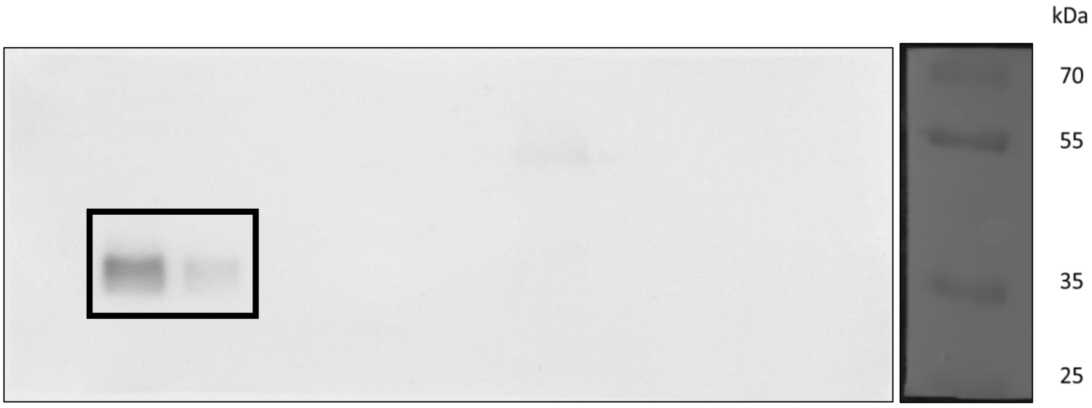

SLC7A5 exp:10s.

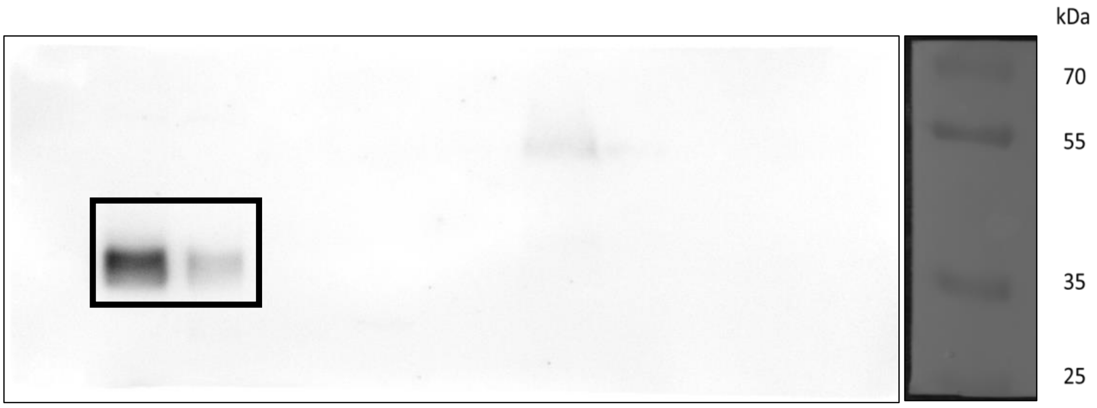

SLC7A5 exp:30s.

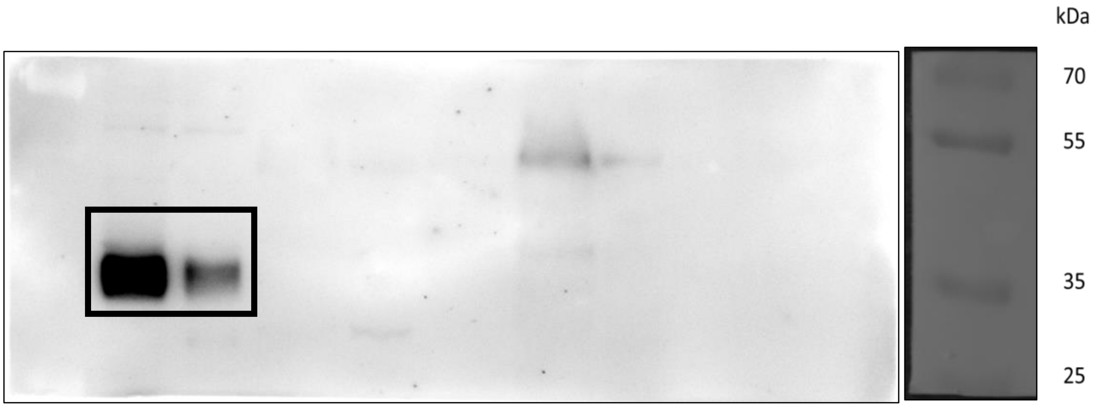

Figure S4. Unprocessed western blot images of actin protein levels in sh-SCR and sh-SLC7A5 BON-1 cells presented in Fig. 5b at different exposure levels.

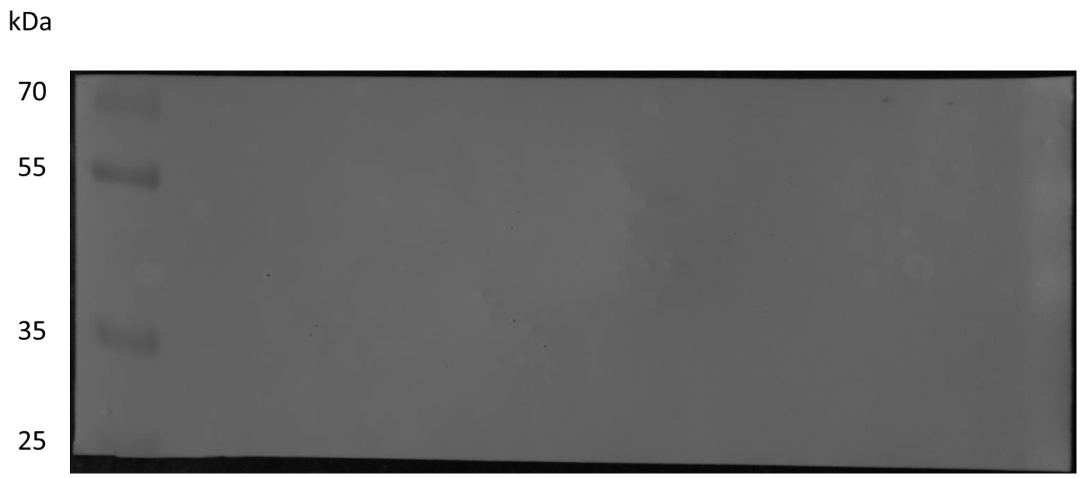

Figure S5. Unprocessed western blot images of SLC7A5 and actin protein levels in sh-SCR and sh-SLC7A5 BON-1 cells presented in Fig. 5b. 25 to 70 kDa membrane photo.
